# P-872. Clinical and Microbiological Trends in *Citrobacter* Bacteremia: A Study from South India

**DOI:** 10.1093/ofid/ofae631.1063

**Published:** 2025-01-29

**Authors:** Rukhsar Abdur Rahim Mulla, Rajalakshmi Ananthanarayanan, Vettakkara Kandy Muhammed Niyas, Febeena Hussain

**Affiliations:** KIMSHEALTH Trivandrum, Thiruvananthapuram, Kerala, India; KIMSHEALTH, Trivandrum, Trivandrum, Kerala, India; KIMSHEALTH, Thiruvananthapuram, Trivandrum, Kerala, India; KIMSHEALTH Trivandrum, Thiruvananthapuram, Kerala, India

## Abstract

**Background:**

*Citrobacter spp* are anaerobic Gram-negative bacilli, accounting for approximately 0.8% of all Gram-negative infections. This study aims to elucidate the clinical manifestations and drug susceptibility in *Citrobacter* infections.

Clinical features of patients with Citrobacter bacteremia and comparison between Citrobacter koseri and other Citrobacter spp

**Figure:**
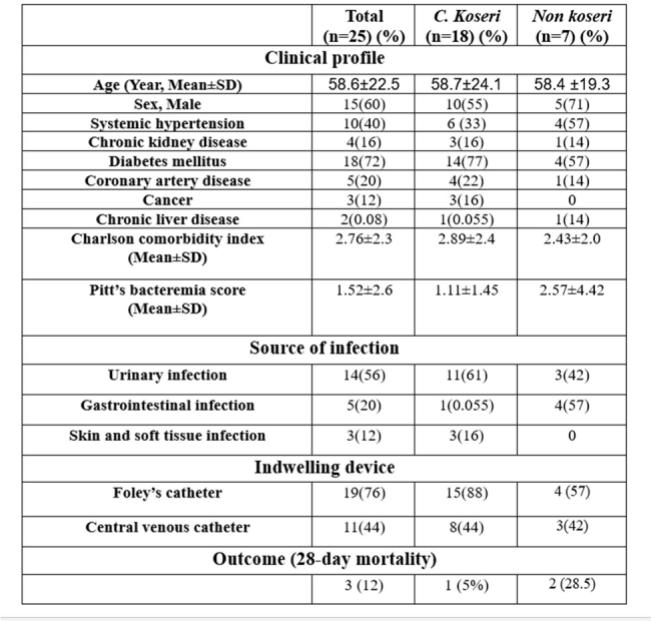

**Methods:**

We conducted a retrospective analysis of *Citrobacter spp*. bacteremia cases from 2016 to 2022. We assessed clinical profile, species, antimicrobial susceptibility and 28-day outcome.

Blood cultures were processed using the BacT/ALERT, Vitek 2 system was employed for bacterial identification and antimicrobial susceptibility testing and reported as per CLSI criteria. Isolates with intermediate susceptibility were classified as non-susceptible.

Comparison of antibiotic susceptibility between C Koseri and other Citrobacter spp

**Figure:**
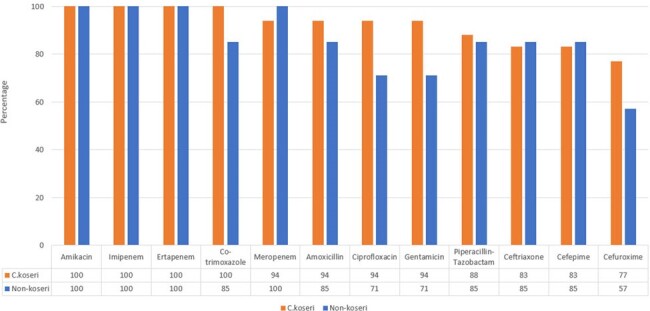

**Results:**

Over the 7 year study period, 25 cases of *Citrobacter* bacteremia were identified, 60% were males. The median age was 52.5 years (IQR: 46 – 75), with 2 pediatric cases.

The annual incidence of *Citrobacter* bacteremia displayed an upward trend, from 12% in 2016 to 24% in 2022. *C. koseri* emerged as the predominant species (72%), followed *by C. freundii* (24%) and *C. farmeri* (4%).

The majority of patients (72%) had diabetes, 16% had chronic liver disease, 12% had malignancy. Risk factors for *Citrobacter* bacteremia included the presence of an indwelling bladder catheter (76%) and a central venous catheter (44%). Urinary tract was the most frequent source (56%), followed by abdominal (20%) and soft tissue (8%); a newborn had meningitis with brain abscess. The mean for Pitt’s bacteremia score was 1.52±2.6. The mean Charlson’s co-morbidity index was 2.76±2.3. Clinical features for the entire cohort and comparative results between *C Koseri* and other *Citrobacter spp* (grouped as non-koseri) is depicted in figure 1.

Ciprofloxacin susceptibility was higher among *C koseri* (94%) as compared to other *spp* (71%). Antibiotic susceptibility for ceftriaxone was almost similar between the 2 groups. Susceptibility rates for other antibiotics and comparison between *C Koseri* and other *spp* are shown in figure 2. All isolates were susceptible to ertapenem, imipenem and amikacin. The overall 28-day mortality rate was 12%.

**Conclusion:**

We found C. koseri more commonly, urinary source as the most common source and device presence as a significant risk factor. Antibiotic susceptibility was better for C. koseri compared to others.

**Disclosures:**

**All Authors**: No reported disclosures

